# Development of GaN-Based, 6.6 kW, 450 V, Bi-Directional On-Board Charger with Integrated 1 kW, 12 V Auxiliary DC-DC Converter with High Power Density

**DOI:** 10.3390/mi15121470

**Published:** 2024-12-02

**Authors:** Alessandro Reali, Alessio Alemanno, Fabio Ronchi, Carlo Rossi, Corrado Florian

**Affiliations:** 1 Department of Electrical, Electronic, and Information Engineering “Guglielmo Marconi”, University of Bologna, 40136 Bologna, Italy; alessandro.reali3@unibo.it (A.R.); carlo.rossi@unibo.it (C.R.); 2 Arca Tecnologie s.r.l., 40026 Imola, Italy

**Keywords:** OBC, totem-pole PFC, DAB converter, phase-shifted full bridge, GaN HEMT, high switching frequency, high power density

## Abstract

Automotive-grade GaN power switches have recently been made available in the market from a growing number of semiconductor suppliers. The exploitation of this technology enables the development of very efficient power converters operating at much higher switching frequencies with respect to components implemented with silicon power devices. Thus, a new generation of automotive power components with an increased power density is expected to replace silicon-based products in the development of higher-performance electric and hybrid vehicles. 650 V GaN-on-silicon power switches are particularly suitable for the development of 3–7 kW on-board battery chargers (OBCs) for electric cars and motorcycles with a 400 V nominal voltage battery pack. This paper describes the design and implementation of a 6.6 kW OBC for electric vehicles using automotive-grade, 650 V, 25 mΩ, discrete GaN switches. The OBC allows bi-directional power flow, since it is composed of a bridgeless, interleaved, totem-pole PFC AC/DC active front end, followed by a dual active bridge (DAB) DC-DC converter. The OBC can operate from a single-phase 90–264 Vrms AC grid to a 200–450 V high-voltage (HV) battery and also integrates an auxiliary 1 kW DC-DC converter to connect the HV battery to the 12 V battery of the vehicle. The auxiliary DC-DC converter is a center-tapped phase-shifted full-bridge (PSFB) converter with synchronous rectification. At the low-voltage side of the auxiliary converter, 100 V GaN power switches are used. The entire OBC is liquid-cooled. The first prototype of the OBC exhibited a 96% efficiency and 2.2 kW/L power density (including the cooling system) at a 60 °C ambient temperature.

## 1. Introduction

In recent years, several institutions worldwide have set regulations on carbon dioxide (CO_2_) emission performance standards aiming to tackle climate change challenges. As a result, the electrification of the global vehicle fleet is expected to continue to grow at a fast rate and to not be limited to passenger, light-duty vehicles but also extended to light commercial vehicles, two- or three-wheelers, buses and trucks [1,2,3]. Electric vehicles (EVs) were introduced into the market in the 2010s with a nominal battery voltage of around 400 V because of the wider availability of automotive-qualified components for that voltage range [4]. Currently, despite the increasing interest to move towards higher DC-link voltages, the 400 V powertrain represents the most mature and suited technology not only for small and medium cars but also for electric sports motorcycles. In fact, higher-voltage batteries enable superior performances in terms of charging speed and global weight reduction, but this is at the expense of higher costs, bulkier housing and a more complex BMS (battery management system) [5,6].

At the present time, a large part—around 80%—of BEVs (battery electric vehicles) are equipped with 400 V batteries, even if the 800 V share is believed to increase up to 40% in the next ten years (from an Infineon internal analysis reported in [7]). Therefore, the development of top-notch 400 V OBCs (on-board chargers) is highly motivated by the EV market trend and is propelled by continuous technology innovations in power electronics.

**Figure 1:**
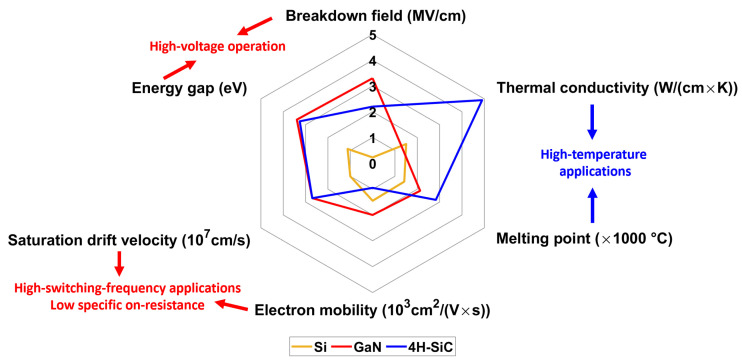
Physical properties of Si, GaN and SiC.

In this study, we propose the design and implementation of a prototype GaN-based, high power density, 450 V, 6.6 kW OBC with a bidirectional power flow capability and the integration of an auxiliary DC-DC converter to connect the 12 V service battery. High compactness and high efficiency requirements are pushing designers to extend the boundary of switching frequencies and adopt WBG (wide-band-gap) semiconductors such as GaN (gallium nitride) and SiC (silicon carbide), which exhibit reduced parasitics and lower switching losses with respect to silicon technology. Looking at the physical properties of semiconductors [8,9] in Figure 1, GaN is particularly suited for high-switching-frequency applications due to its excellent electron mobility and saturation velocity, which also ensure a low channel resistance.

From the first release of E-mode GaN-on-silicon in 2009, GaN power switches have come across almost two decades of innovations [10]. At the present time, automotive-grade, 650 V, 25 mΩ devices represent the best technology in the market for medium-voltage-class applications. Recently, several semiconductor companies have released or announced GaN-power ICs which also feature the monolithic integration of gate drivers [11,12] and further R&D activities are exploring the development of two-gate monolithic bidirectional switches with a bipolar voltage-blocking capability and bidirectional current control, which are expected to be the next breakthrough in power electronics [13,14]. However, at the moment, GaN HEMT discrete devices are the most mature and available technology provided by several semiconductor suppliers in the market.

**Figure 2:**
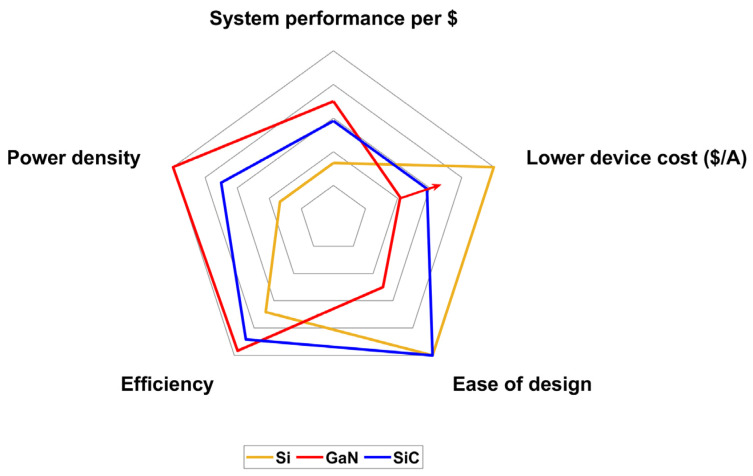
Converter design FOMs exploiting Si, GaN and SiC devices, from [17].

Nevertheless, GaN adoption comes along with some design challenges resulting from the very high slew rates of voltage and current at commutations which impose particular attention on the parasitics of PCB layout as well as of passive components [15,16]. As depicted in Figure 2, from [17], device costs can also be a limiting factor, although GaN is believed to become less expensive in the future, benefiting from economies of scale.

By the way of comparison, some commercially available OBC designs in the medium power range are reported in Table 1. The performances of the proposed design are state-of-the-art, considering that it includes the additional LV DC-DC converter, the cooling plate and the enclosure. Moreover, this is the first prototype that will undergo further volumetric occupancy optimization in its final release. 

In the following, Section 2 details the development of the prototype on-board charger, addressing the PFC (Section 2.1), DAB (Section 2.2) and PSFB (Section 2.3) design choices. In Section 2.4, the simplified control architecture is briefly explained, and, finally, in Section 3, the OBC implementation and preliminary validation measurements are presented.

## 2. OBC Design

**Figure 3:**
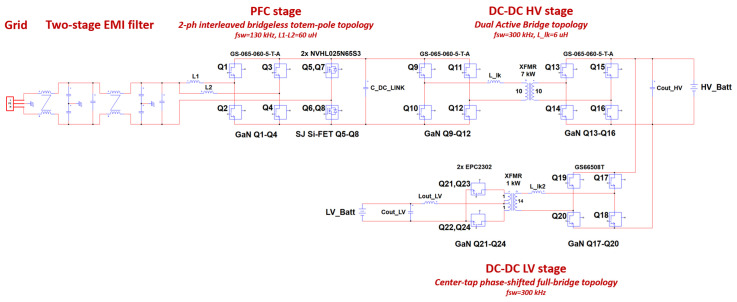
OBC system’s topology.

The OBC under design is required to comply with several AC charging markets (above all from the EU, the USA, China and Japan). Hence, the PFC (power factor correction) AC/DC stage is capable of handling a wide range (90–264 Vrms, 50/60 Hz) of AC mains supplies. A second DC-DC HV stage is used to regulate the battery voltage and current, both in G2V (grid-to-vehicle) and V2G/V2L (vehicle-to-grid/vehicle-to-load) operation modes with a battery voltage ranging between 200 V and 450 V. An auxiliary DC-DC LV stage is used to connect the 12 V service battery with bidirectional capability, charging the low-voltage battery from the HV DC rail as well as providing features such as limp home and the inverter DC-link capacitor’s precharge from the low-voltage battery. Table 2 lists the OBC’s main specifications, while Figure 3 displays the designed system’s topology. A PSIM 2022.3 software package (now from Altair Engineering Inc., Troy, MI, USA) was used to validate the design choices by the means of electrical and thermal simulations.

### 2.1. PFC Converter Design

The PFC converter is realized in a 2-ph, interleaved, bridgeless, totem-pole topology, which offers notable advantages over the conventional boost or the 2-ph bridgeless circuits [21]. First of all, the elimination of diode bridge losses allows the improvement of the efficiency from 97–98% to 99% or higher, ensuring bidirectional capability as well. Furthermore, it benefits from lower part counts, enabling a higher power density and lower BOM cost. Not least when GaN devices are used in the half-bridge configuration, the inherent absence of a parasitic body-diode guarantees zero reverse recovery loss upon turn-on (Q_rr_ = 0), making it possible to operate in CCM (continuous conduction mode) even at high power levels with lower harmonics (higher PF quality) and a lower rms current (higher efficiency), as opposed to DCM/CrCM (discontinuous conduction mode/critical conduction mode), which are implemented to avoid body-diode conduction when Si mosfets are used instead [21,22].

The automotive-grade, top-side-cooled, 650 V, 25 mΩ GS-065-060-5-T-A GaN HEMT device from GaN Systems (now Infineon Technologies) was selected to be used in the two HF (high-frequency) half-bridges of the PFC, as well as in the DC-DC HV converter. Its main datasheet parameters are listed in Table 3. 

Super-junction (SJ) Si mosfets (650 V, 19.9 mΩ) are used in the LF leg, ensuring bidirectional capability and synchronous rectification with higher efficiency with respect to IGBTs or FRDs (fast recovery diodes). Operating at line frequency, they exhibit negligible switching losses. The usage of two Si mosfets in parallel per switch enables similar conduction losses compared to the GaN HEMTs in the two-phase interleaved HF legs.

Regarding the boost power inductors, the combination of two-phase interleaving, CCM operation and high switching frequency (f_sw_ = 130 kHz) enables the best compromise between distortion and power density, using a significantly reduced inductance value (L1, L2 = 60 µH) with respect to traditional silicon-based PFC circuits (typically PFC inductors in the range 300–500 µH). The inductance value of each PFC channel can be determined as follows [12]:

(1) $$L=\frac{V_{out}\;/\;2}{k_{ripple}\cdot \sqrt{2}\cdot I_{L,rms}\cdot 2\cdot f_{sw}}$$

where *k_ripple_* < 1 is the ripple coefficient and *I_L,rms_* is the rms current of the PFC channel.

A PSIM simulator exploits the so-called thermal module model [23] in order to calculate conduction and switching losses on the basis of several look-up table datasets (first and third quadrant characteristics as well as E_on_ and E_off_ values as functions of V_GS_, V_DS_, I_DS_, R_G_ and T_j_ of device). This approach enables us to avoid the large computational effort of Pspice-like simulators which calculate power dissipation based on the integration of V_DS_ and I_DS_ waveforms. Moreover, the thermal model allows us to retrieve the junction and case temperature delta due to the power dissipation by the means of the junction–case thermal impedance and the thermal resistance of the connection between the TIM (thermal interface material) and the liquid cold plate. Furthermore, PSIM simulations take into account accurate models of the passive components (in Table 4 for the PFC converter).

Custom-designed PFC inductors exploit a gapped ferrite core, ensuring 5 A of margin between the peak current and the saturation point. Furthermore, windings in Litz wire guarantee an AC resistance which is close to the DC resistance value. A really compact size is achieved: each PFC inductor is encapsulated in a 46.1 (L) × 38.6 (W) × 46 (H) [mm] potting box.

**Figure 4:**
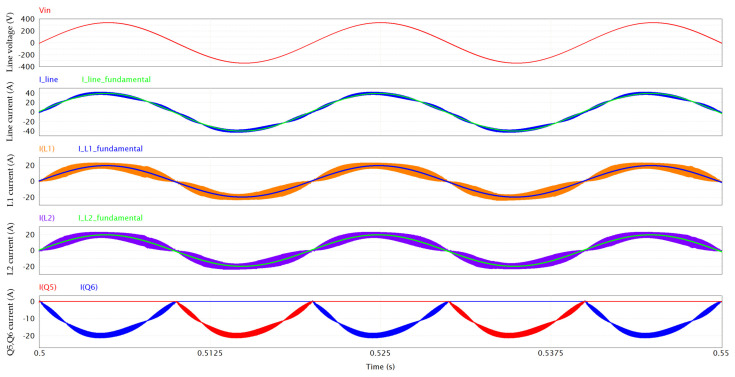
PFC waveforms, including, from top to bottom, line voltage, line current and its fundamental component (50 Hz), inductor L1 current and its fundamental component (50 Hz), inductor L2 current and its fundamental component (50 Hz), Q5 and Q6 (LF leg) currents.

Starting from the Figure 4, PFC simulation results are reported hereafter. The following operating conditions are assumed: V_in_ = 240 Vrms; f_line_ = 50 Hz; L1 = L2 = 60 µH; V_out_ = 400 V; P_out_ = 6.6 kW; f_sw_ = 130 kHz; dead time = 100 ns; T_amb_ = 60 °C (the maximum temperature of the cooling plate). The GaN devices are driven with V_GS_ = 6/−3 V and R_G_ = 10/2 Ω, whereas the Si mosfets are driven with V_GS_ = 10/0 V and R_G_ = 2 Ω.

**Figure 5:**
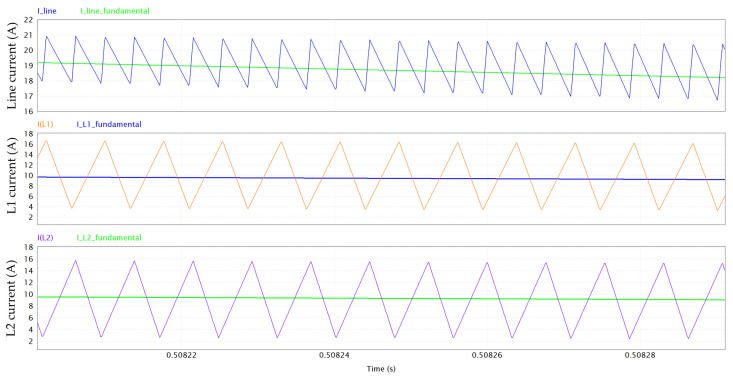
Magnification of ripple of line current (at 260 kHz) and of L1/L2 current (at 130 kHz).

Figure 5 provides a magnification of the inductor ripple and the line current ripple, which is enhanced by the interleaved operation. Despite the very low value of inductance (60 µH), the combination of a high switching frequency and interleaving results in a computed THD of 6%. From the simulation results, the PF (power factor) turns out to be 0.996.

**Figure 6:**
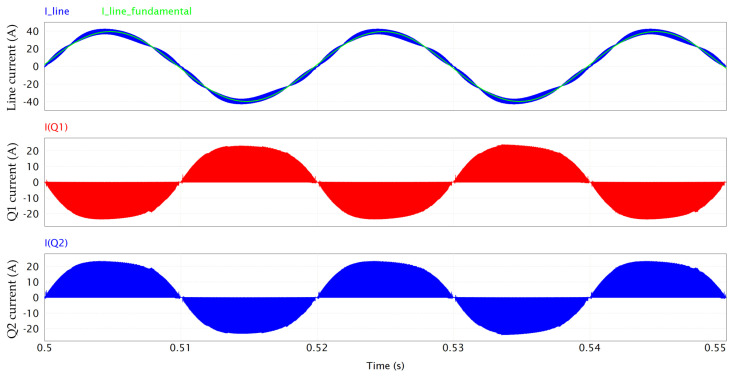
Q1-Q2 HF current conduction.

Figure 6 shows the HF currents conducted by the first GaN half-bridge Q1-Q2.

**Figure 7:**
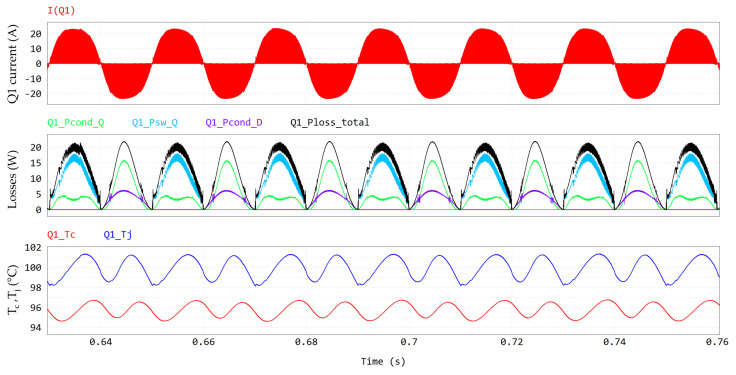
Q1 losses, including, from top to bottom, Q1 current, conduction losses (in green), switching losses (in cyan), third-quadrant losses (in violet), total losses (in black) and case (Tc) and junction (Tj) temperatures.

**Figure 8:**
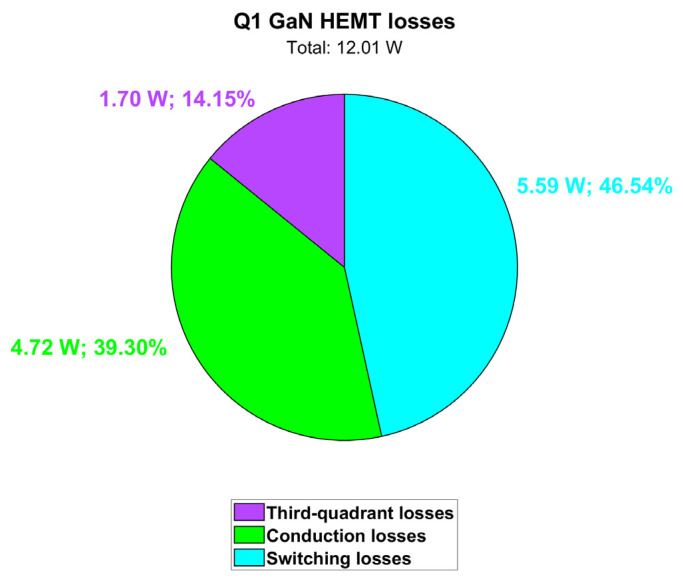
Contributions of Q1 power losses (@ T_amb_ = 60 °C).

As observed before, the exploitation of PSIM thermal models enables the accurate computation of transistor losses and their different contributions. Power losses contributions for Q1 are displayed in Figure 7 along with case and junction temperatures. The waveforms of Figure 7 are at a stable thermal regime. Nonetheless, the variation in the junction temperature and the power dissipation within the slow 50 Hz period can be appreciated. It can be observed that in this nominal full power condition (P_out_ = 6.6 kW) at the maximum cooling plate temperature (T_amb_ = 60 °C), the GaN HEMT channel temperature (T_j_) is still safely far from its maximum rating of 150 °C. By computing the average values of dissipated power, the pie chart in Figure 8 can be obtained, showing the power losses distribution. Third quadrant losses occur during dead time, when the GaN HEMT has a diode-equivalent behavior with a forward voltage equal to −V_GS(th)_ − |V_GS,off_| − R_ds,rev_ · I_SD_ ≈ −5 V [24]. We can also consider these losses conduction losses. It is interesting to observe that there is an almost even distribution between the conduction and switching losses, meaning that the selected switching frequency represents a very good compromise between the switching losses and shrinking of the inductor size.

Similarly to the CCM boost PFC, the capacitance value of the DC-link capacitor is determined by voltage ripple and hold-up time requirements [25]:

(2) $$C_{DC-link}\geq \;\frac{P_{out}}{V_{out}\cdot 2\cdot \pi \cdot f_{line}\cdot {∆V}_{pk-pk}}$$

(3) $$C_{DC-link}\geq \;\frac{2\cdot P_{out}\cdot t_{hold-up}}{V_{out}^{2}-V_{out,min}^{2}}$$

Based on Equations (2) and (3), at least 1.2 mF are necessary to guarantee a voltage ripple of 44 V_pk-pk_ when P_out_ = 6.6 kW and V_out_ = 400 V, as well as a hold-up time of 10 ms with the minimum acceptable output voltage V_out,min_ ≈ 220 V.

The total current through the output capacitance C_DC-LINK_ has two main components: a dominant low-frequency (LF) component at twice the line frequency and a high-frequency component at the switching frequency and its harmonics. As observed in [25,26], the low-frequency rms component can be calculated as

(4) $$I_{LF,rms}=\frac{P_{out}}{V_{out}\cdot \sqrt{2}}=\frac{I_{out}}{\sqrt{2}}=\frac{16.5\;Arms}{\sqrt{2}}=11.67\;\mathrm{Arms}$$

while the high-frequency rms component in the case of 2-ph interleaving can be approximated as follows [26]:

(5) $$I_{HF,rms,2-ph}=\sqrt{mt\;\left(\frac{3}{2}\cdot \frac{P_{in}^{2}}{V_{out}^{2}}-\frac{64}{15\pi }\cdot \frac{P_{in}^{2}\cdot V_{in\_pk}}{V_{out}^{3}}\right)+ct\left(\frac{16}{3\pi }\cdot \frac{P_{in}^{2}}{V_{in\_pk}\cdot V_{out}}-\frac{3}{2}\cdot \frac{P_{in}^{2}}{V_{out}^{2}}\right)}$$

where *mt* and *ct* are the coefficients for the linearization of the correction factor K_MS_(t).

(6) $$K_{MS}\left(t\right)=mt\;\frac{V_{in\_pk}}{V_{out}}\sin\left(\omega t\right)+ct$$

*mt* = −1.2 and *ct* = 0.6 when the on-time duty cycle d_on_(t) = 1−(V_in_pk_/V_out_)∙sin(*ω*t) > 0.5, whereas *mt* = 1.2 and *ct* = −0.6 when d_on_(t) < 0.5. In our case, under the mentioned operating conditions, from (5), the DC-link HF component turns out to be equal to

(7) $$I_{HF,rms,\;2-ph}=5.70\;\mathrm{Arms}$$

**Figure 9:**
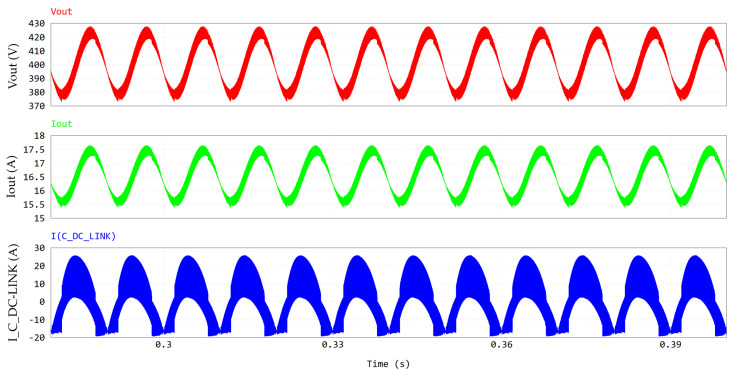
PFC waveforms at the output section: V_out_, I_out_, I_C_DC-LINK_.

The total rms current through the DC-link capacitors can be then calculated as

(8) $$I_{C,\;DC-link}=\sqrt{I_{LF,rms}^{2}+I_{HF,rms,\;2-ph}^{2}}=\sqrt{11.67^{2}{\mathrm{Arms}}^{2}+5.70^{2}{\mathrm{Arms}}^{2}\;}=12.99\;\mathrm{Arms}$$

which is consistent with the simulation result reported in Table 5. The DC-link capacitor bank is realized with three electrolytic capacitors in parallel (Kemet ALA7DA391CF500, 500 V, 390 µF). Figure 9 shows an output voltage ripple of 45 V_pk-pk_.

**Figure 10:**
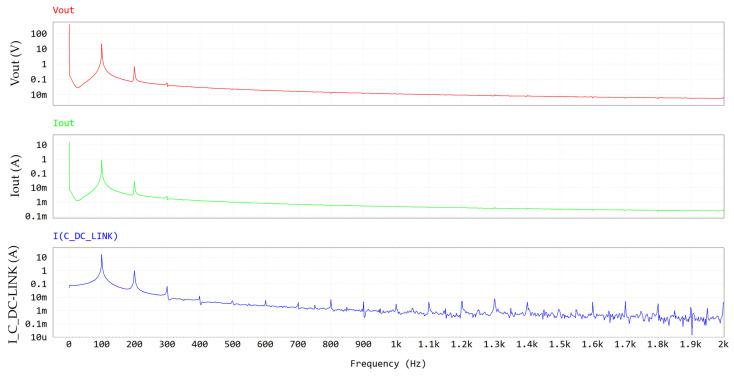
FFT (0–2 kHz) of PFC waveforms at output section. Y-axis is in log scale.

**Figure 11:**
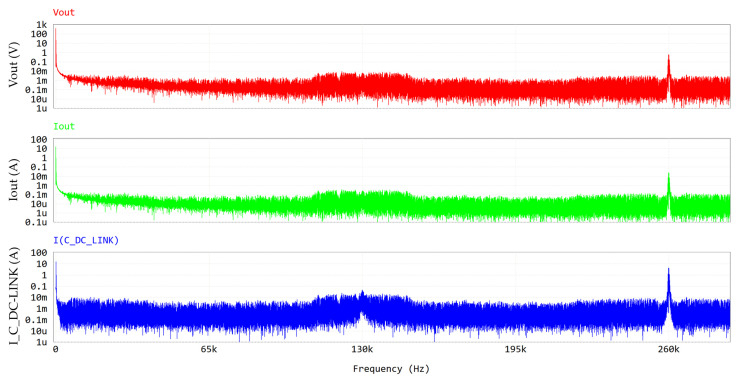
FFT (0–280 kHz) of PFC waveforms at output section. Y-axis is in log scale.

Figure 10 and Figure 11 display the frequency spectra of V_out_, I_out_ and I_C_DC-LINK_, allowing the evaluation of their harmonic content (see Table 6).

Table 6 reports the amplitude of the LF component of the DC-link current (16 Apk = 11.31 Arms) which is close to the DC component of the output current (16.5 Apk ≈ 16.5 Arms), justifying Equation (4). The HF component at twice the switching frequency is displayed in Figure 11.

The ESR of electrolytic capacitors decreases with temperature, for ALA7DA391CF500 can be estimated at 250 mΩ at 70 °C. Then, the losses of the DC-link capacitor bank can be calculated as

(9) $$P_{DC-link\;cap\;bank}=\frac{ESR}{3}\cdot I_{C,DC-LINK}^{2}=\frac{250\;mΩ}{3}{\cdot 13.32}^{2}{\mathrm{Arms}}^{2}=14.79\;W$$

where I_C,DC-LINK_ is the total ripple current of the DC-link capacitors from Table 5.

The DC resistance of the power inductors is equal to 22 mΩ. The inductor copper losses related to the DCR can be computed as

(10) $$P_{L.DCR}=DCR\cdot I_{L,rms}^{2}=22\;mΩ{\cdot 14.27}^{2}{\mathrm{Arms}}^{2}=4.48\;W$$

**Figure 12:**
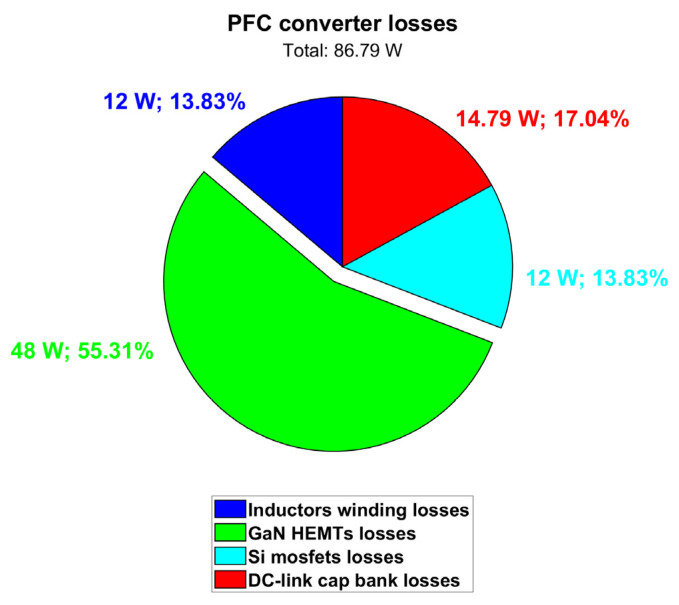
PFC converter power loss contributions (@ T_amb_ = 60 °C).

Simulation performed through ANSYS software resulted in total winding losses of 6 W, also taking into account the contribution of eddy currents (i.e., the AC resistance). Table 7 and Figure 12 summarize the PFC simulation results. Average temperatures and power losses at regime are considered. The total dissipated power is quantified in 86.79 W and PFC efficiency turns out to be 98.70%. This performance is in the worst-case condition of 60 °C coolant temperature.

**Figure 13:**
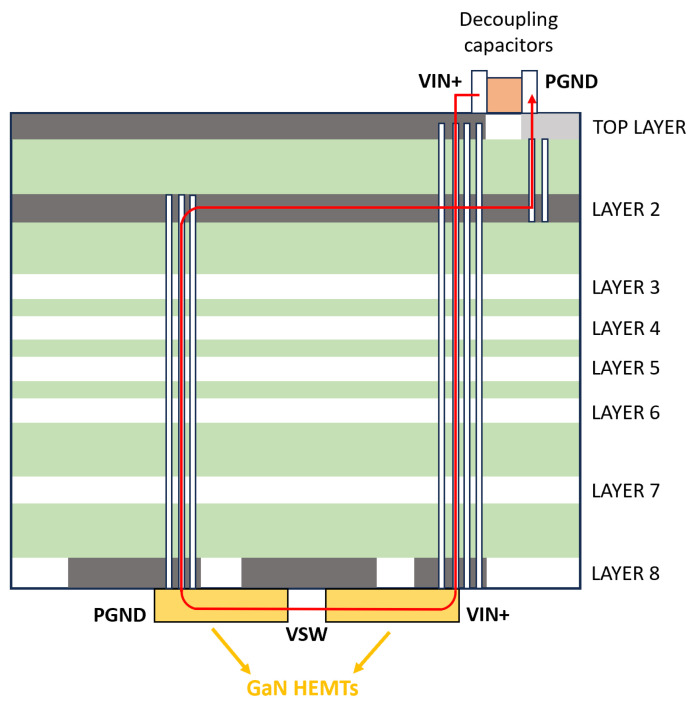
Flux-canceling traces with top-side-cooled devices in an 8-layer PCB. Layer 2 is used as a ground return. The thickness of the entire layer stack-up is 2060 µm in our case [16].

These computed performances can be achieved by the actual implementation of the converter provided that optimal layout of the PCB is designed. Indeed, the fast-switching behavior of GaN devices imposes very high slew rates of voltage and current in commutations (up to hundreds of volts per nanosecond and ten amps per nanosecond). Therefore, it is fundamental to precisely assess, minimize and compensate any inductive or capacitive parasitics of the circuit. One of the major concerns is related to driver and power loops, i.e., the gate-source and drain-source loops of the device. The GS-065-060-5-T-A embedded package ensures ultra-low stray inductances with respect to the traditional wire-bonded QFNL (Quad Flat No-Lead) or TO (Transistor Outline) packages, at the expense of higher costs. Furthermore, an optimal PCB layout—with a wise driver loop and power/ground planes arrangement along with an accurate selection of passive components and mechanical connectors—plays an essential role. For this aim, the very compact layout of the GaN switching leg was designed by implementing a very compact driving loop and also exploiting magnetic flux cancelation in the power loop, with top-side-cooled GaN HEMTs on the bottom layer (to be directly connected to the cold plate), while drivers and low-parasitic, high-current decoupling capacitors are placed on the top layer (see Figure 13). The top-side-cooled GS-065-060-5-T-A GaN HEMT does not provide a separate Source Sense pin (as opposed to the bottom-side-cooled counterpart GS-065-060-5-B-A). However, for the same purpose, a Kelvin connection at the side of Source pad was routed, separating the drive return and the power ground, minimizing the common source inductance and thus the noise coupling between the two loops [24]. Also, Allegro AHV85110 isolated single-channel drivers that feature in-package micro-transformer and 2 A/4 A of source/sink current were used to provide an optimal GaN HEMTs driving in a very compact form factor. The optimization of the layout of the GaN switching leg was also supported by EM simulations as detailed in [16].

The low-frequency switching leg (with super-junction silicon mosfets) is not critical and is implemented with a traditional PCB layout exploiting through hole connection of TO-247 package devices, driven by a Texas Instruments UCC21530BQDWKRQ1 4A/6A isolated dual-channel driver.

Some pictures of the OBC will be shown later in Section 3.

### 2.2. DAB Converter Design

In two-stage OBC designs, the PFC converter is followed by a DC-DC HV stage to regulate the battery charging process. The exploitation of bidirectional devices and suitable control algorithms enables V2G/V2L operation modes as well. High efficiency and high power density requirements imposed by automotive players are leading power electronics designers to the implementation of an increasingly high switching frequency [27]. Since in hard-switching topologies power losses in commutations are proportional to the switching frequency, the adoption of power devices in WBG technology is necessary to minimize losses and achieve high-efficiency (>96%) converters. For OBCs in the medium-voltage/medium-power class, 650 V, 25 mΩ GaN HEMTs are an excellent choice. Further enhancements in efficiency (>98%) can be reached by the means of ZVS (zero-voltage switching) topologies which can ensure almost negligible switching losses [28].

DC-DC converter specifications (in Table 2) include a wide output voltage range (200–450 V). Galvanic isolation is also required. The most promising topologies are the resonant CLLC and the dual active bridge. The latter has been preferred due to the simplicity of its design and control scheme; in an SPS (single-phase shift) modulation, the power flow is controlled by regulating the voltage applied to the primary series inductor by simply adjusting the time displacement (phase shift) among the gate signals of the two full bridges, avoiding a non-linear relationship between the gain and the load condition that exists in a CLLC circuit, where the operating switching frequency at a small Q (quality factor) can reach very high values [29,30].

**Figure 14:**
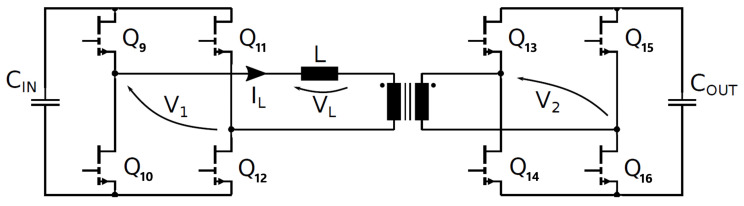
DAB DC-DC converter schematic.

With reference to the schematic in Figure 14, the relationship between the output power and the phase shift, *ϕ*, is equal to the following [31]:

(11) $$P=\frac{nV_{1}V_{2}}{2\pi^{2}f_{sw}L}\;\phi \;\left(\pi \;-\;\left|\phi \right|\right)$$

where *n = N*_2_*/N*_1_ is the transformer turns ratio and − π/2<ϕ<π/2. The absolute maximum power is obtained for *ϕ = π / 2*:

(12) $$\left|P_{max}\right|=\frac{nV_{1}V_{2}}{8f_{sw}L}$$

The designed DAB converter is composed of two full bridges of GS-065-060-5-T-A GaN HEMTs driven by the Allegro single-channel isolated driver AHV85110, which features a Power-Thru Integrated Isolated Bias Supply. The PCB layout of each switching leg is the same as described for the PFC section, which guarantees optimal performances by minimizing parasitics.

The selected switching frequency is 300 kHz, which allows the development of a very compact custom transformer. DAB ZVS boundaries depend on the total energy stored in the series inductor and can be calculated as reported in [29,31]. A series inductance of 6 µH was selected to ensure a wide ZVS region and provide an output power of 6.6 kW when V_in_ = V_out_ = 400 V; f_sw_ = 300 kHz; *n* = 1; and the phase shift = 33°.

The series inductance is represented by the leakage inductance (6 µH) of the custom-designed DAB transformer (*n* = 1) without the need for an external shim inductor. This is crucial to minimize the dimensions of the converter; the DAB transformer is encapsulated in a 65.19 (L) × 47.51 (W) × 46.90 (H) [mm] potting box. Regarding the mixed-type capacitor bank formed by the electrolytic low-frequency capacitor C_LF_ and ceramic high-frequency capacitor C_HF_, it is of paramount importance in WBG applications to minimize the involved parasitic inductance, especially the ESL of the LF capacitor and inductance of the connection structure, otherwise antiresonance issues at high frequencies may arise. A maximum overall inductance of two digits of nH should be met.

The DAB’s passive components are listed in Table 8.

**Figure 15:**
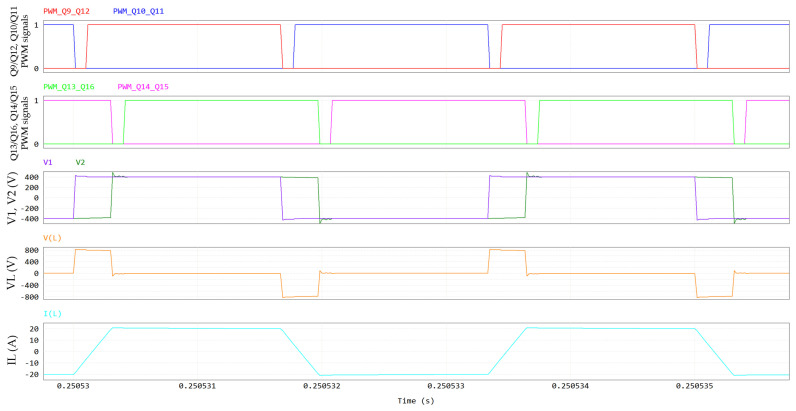
DAB waveforms, including, from top to bottom, PWM signals of Q9-Q12 and Q10-Q11, PWM signals of Q13-Q16 and Q14-Q15, primary (V1) and secondary (V2) voltages, series inductor voltage V_L_ and series inductor current I_L_.

**Figure 16:**
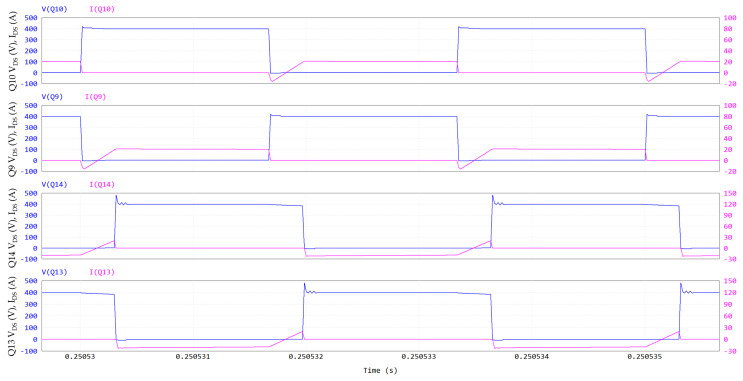
DAB ZVS turn-on: V_DS_ and I_DS_ of Q10, V_DS_ and I_DS_ of Q9, V_DS_ and I_DS_ of Q14, V_DS_ and I_DS_ of Q13. Y-axis of drain-source voltage is on left, and Y-axis of drain-source current is on right.

The DAB waveforms (400 V/400 V; 6.6 kW; 300 kHz; V_GS_ = 6/−3 V; R_G_ = 10/2 Ω; dead time = 100 ns; T_amb_ = 60 °C) simulated through PSIM are displayed in Figure 15 and Figure 16. The names of the electrical quantities correspond to the labels in Figure 14. In an SPS modulation, switches on the same diagonal (Q9-Q12, Q10-Q11, Q13-Q16, Q14-Q15) are ON/OFF for half a period and share the same gate signal with a 50% duty cycle. Figure 15 and Figure 16 show that when Q10-Q11 are turned off, the negative inductive current I_L_ charges the C_oss_ of Q10-Q11 to 400 V + V_f_ and discharges the C_oss_ of Q9-Q12 to -V_f_, where V_f_ ≈ 5 V is the diode-equivalent forward voltage of GaN in reverse conduction. Then, V1 commutes to 400 V + 2 V_f_, VL commutes to 800 V + 2 V_f_ and the series inductor is charged. Similarly, when Q14-Q15 are turned off, V2 commutes to 400 V + 2 V_f_, VL commutes to −2 V_f_ (since Q9-Q12 stopped their reverse conduction) and the series inductor is slowly discharged. In the second half of the period, Q9-Q12 are turned off (V1 toggles to −400 V −2 V_f_ and VL toggles to −800 V −2 V_f_), followed by Q13-Q16 (V2 toggles to −400 V −2 V_f_ and VL toggles to +2 V_f_).

It is noteworthy that GaN HEMTs experience a ZVS turn-on; for instance, during the dead time that follows Q10-11 turn-off, the negative inductive current I_L_ discharges the C_oss_ of Q9–12 to −V_f_. Hence, Q9-Q12 are in reverse conduction in third quadrant, acting as equivalent free-wheeling diodes (the gate is OFF). Then, when the PWM signal of Q9-Q12 goes ON, they are forward-biased with an almost-null drain-source voltage, which further decreases, in absolute terms, from −V_f_ to −V_DS,ON_, leading to negligible turn-on losses. The negative sign of V_DS_ is due to the fact that Q9-Q12 have opposite polarity when I_L_ is negative (see Figure 14).

In Figure 15 and Figure 16, all the DAB switches experience a ZVS turn-on at V_out_ = 400 V and P_out_ = 6.6 kW. Since they are piloted per diagonal lines, Figure 16 is representative for all eight switches.

**Figure 17:**
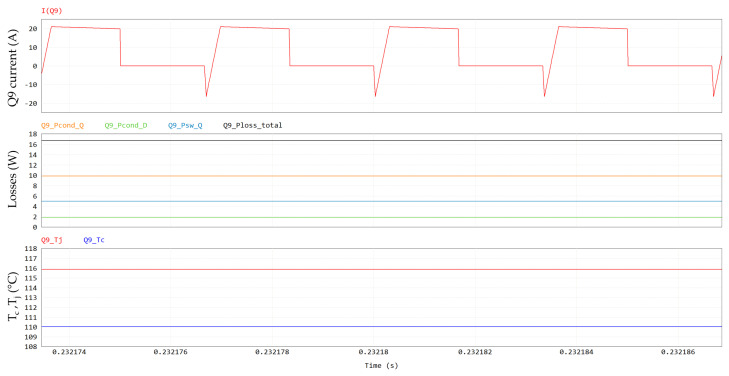
Q9 losses, including, from top to bottom, Q9 current, conduction losses (in orange), third-quadrant losses (in green), switching losses (in light blue), total losses (in black) and case and junction temperatures.

**Figure 18:**
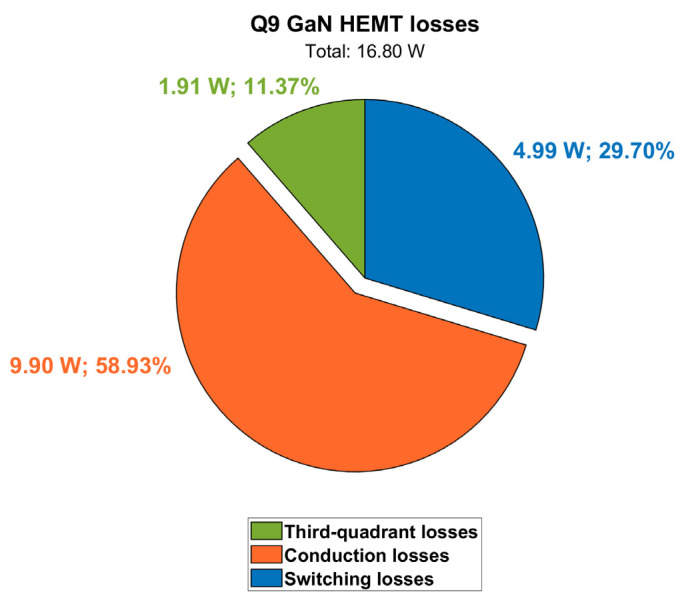
Contributions of Q9 power losses (@ V_out_ = 400 V, P_out_ = 6.6 kW, T_amb_ = 60 °C).

Power losses contributions at a steady-state for Q9 are displayed in Figure 17, along with case and junction temperatures. It can be noted that at full-power condition (P_out_ = 6.6 kW) and at the maximum cooling plate temperature (T_amb_ = 60 °C), the GaN HEMT channel temperature (T_j_) reaches 116 °C, which is still safely far from its maximum rating of 150 °C. The pie chart in Figure 18 shows the weights of the different distributions.

In [16], the authors extensively discussed the details of the ZVS turn-on and turn-off commutations of GS-065-060-5-T-A GaN devices in the described DAB converter prototype, exploiting the Pspice non-linear dynamic model of the transistor and distinguishing between the channel current and parasitic capacitance (*C_gs_*, *C_gd_*, *C_ds_*) currents, also taking into account the parasitics of the designed PCB. Simulations have made it possible to underline the absence of the Miller plateau both in turn-on and turn-off commutations [32], as well as a Miller voltage below the threshold at turn-off in case of a strong driver [28], when GaN devices experience an almost ZVS turn-off with nearly negligible turn-off losses [33]. This denotes that PSIM simulations overestimate the switching losses of the device in case of ZVS behavior, since the thermal model computes power dissipations based on the pre-commutation values of V_DS_ and I_DS_ waveforms. This underlines the need for accurate analyses of the non-linear dynamic behavior of GaN devices in order to achieve precise assessments of power losses and of corresponding thermal design. In this study, we take the PSIM thermal model losses as valid, considering them a worst-case scenario for the thermal budget of the cooling system design.

Other main losses are related to the DAB transformer. Primary/secondary DC resistances are equal to 9.4 mΩ, leading to about (3.85 + 3.85) W of copper losses. Core losses (PQ60-42Z Ferrite, DMR95, with gap of 0.1 mm) can be estimated as 10 W.

Taking into consideration only the OBC DC-DC stage under examination, the rms current of electrolytic capacitors are quantified as 3.54 Arms for the DC-link section and 1.47 Arms for the output section. Then, the losses of the electrolytic capacitors can be calculated as follows:

(13) $$P_{DC-link\;cap\;bank}=\frac{ESR}{3}\cdot I_{C,DC-LINK}^{2}=\frac{250\;mΩ}{3}{\cdot 3.54}^{2}{\mathrm{Arms}}^{2}=1.04\;W$$

(14) $$P_{output\;cap}=ESR\cdot I_{C,out}^{2}=250\;mΩ{\cdot 1.47}^{2}{\mathrm{Arms}}^{2}=0.54\;W$$

**Figure 19:**
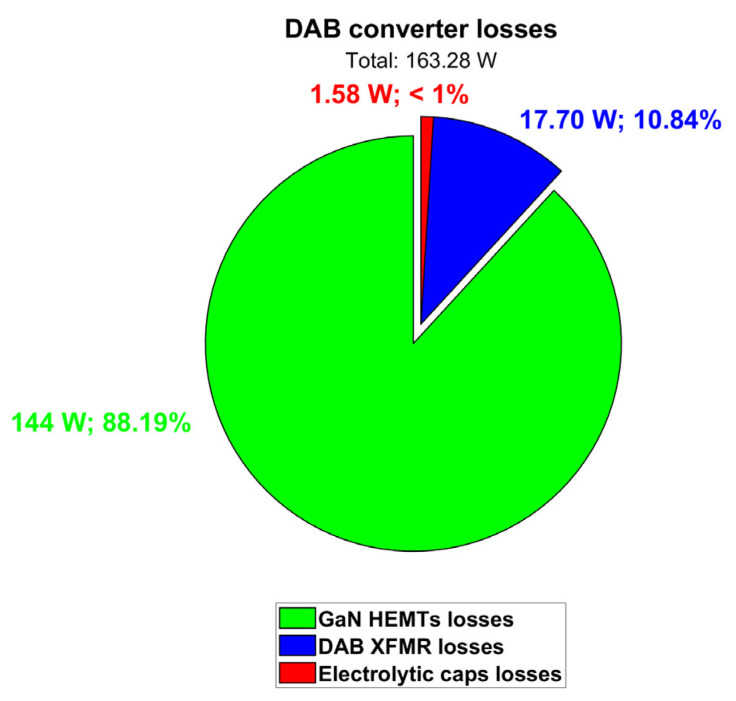
Contributions of DAB converter losses (@ V_out_ = 400 V; P_out_ = 6.6 kW; T_amb_ = 60 °C).

Taking all these loss contributions into account, the total dissipated power is quantified as 163.28 W, and the DAB efficiency turns out to be 97.59%. Table 9 and Figure 19 summarize the DAB simulation results at V_out_ = 400 V and P_out_ = 6.6 kW.

In the following, simulation results at V_out_ = 250 V, I_out_ = 16.5 Arms and P_out_ = 4.125 kW are provided as further evidence of the design’s success; a high efficiency value (>96%) was achieved under these operating conditions as well (the coolant temperature was always at the worst-case condition of 60 °C), also proving high performance at low battery voltage values with respect to the nominal condition (400 V).

**Figure 20:**
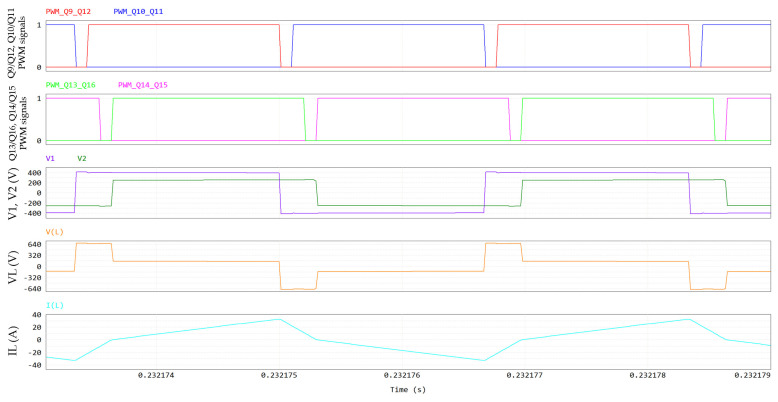
DAB waveforms at V_out_ = 250 V and P_out_ = 4.125 kW, including, from top to bottom, PWM signals of Q9-Q12 and Q10-Q11, PWM signals of Q13-Q16 and Q14-Q15, primary (V1) and secondary (V2) voltages, series inductor voltage V_L_ and series inductor current I_L_.

**Figure 21:**
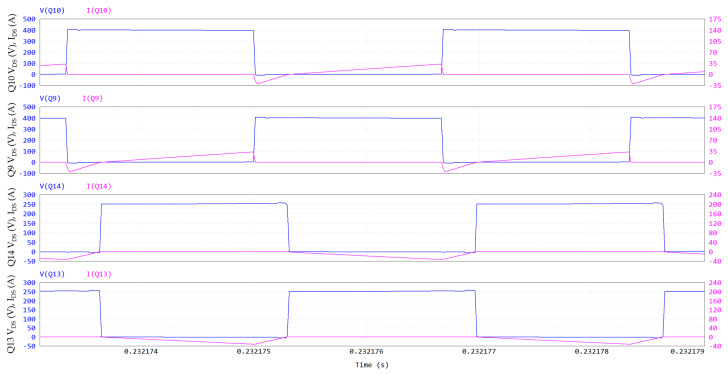
DAB Q9 and Q10 ZVS turn-on (@ V_out_ = 250 V and P_out_ = 4.125 kW): V_DS_ and I_DS_ of Q10, V_DS_ and I_DS_ of Q9, V_DS_ and I_DS_ of Q14, V_DS_ and I_DS_ of Q13. Y-axis of drain-source voltage is on left, and Y-axis of drain-source current is on right.

**Figure 22:**
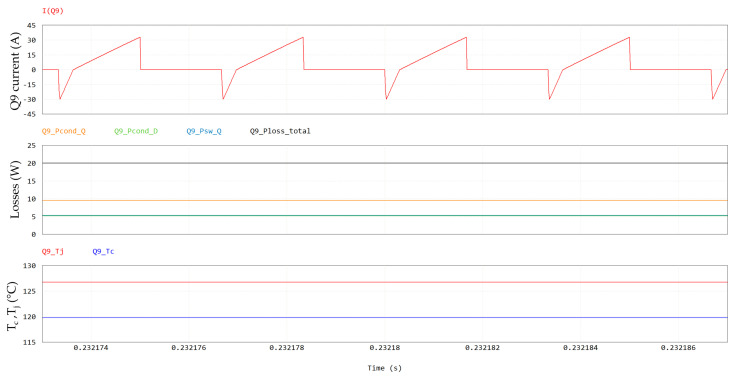
Q9 losses at V_out_ = 250 V and P_out_ = 4.125 kW: Q9 current, conduction losses (in orange), third-quadrant losses (in green), switching losses (in light blue), total losses (in black) and case and junction temperatures.

**Figure 23:**
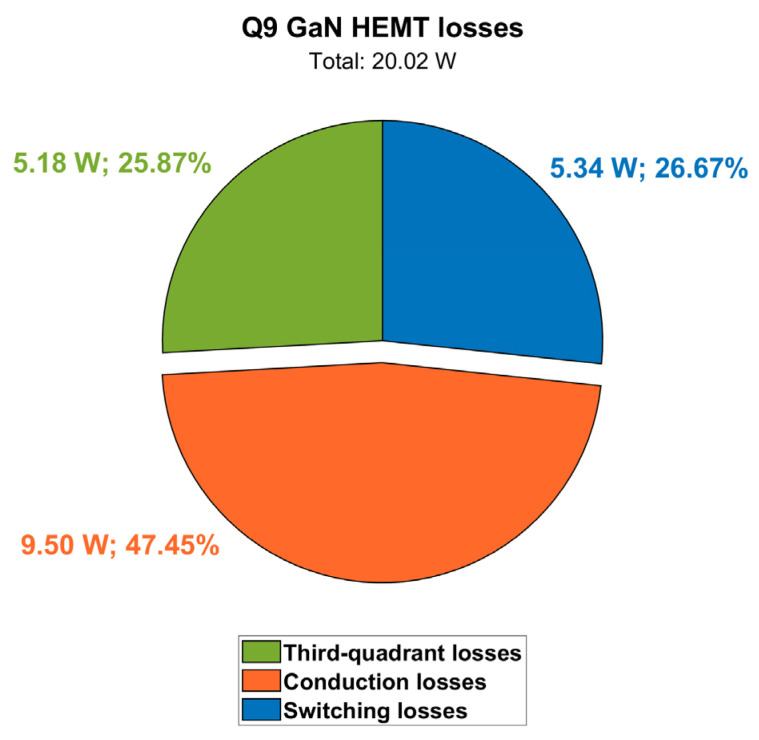
Contributions of Q9 power losses (@ V_out_ = 250 V; P_out_ = 4.125 kW; and T_amb_ = 60 °C).

**Figure 24:**
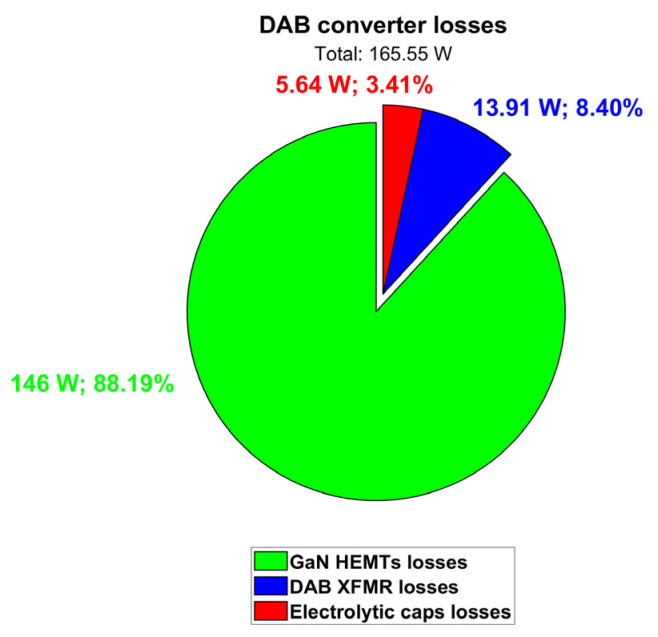
Contributions of DAB converter losses (@ V_out_ = 250 V; P_out_ = 4.125 kW; and T_amb_ = 60 °C).

In Figure 20 and Figure 21, Q9 and Q10 also experience a ZVS turn-on at V_out_ = 250 V and P_out_ = 4.125 kW. Power losses’ contributions for Q9 are displayed in Figure 22 along with case and junction temperatures. A pie chart of Q9’s power losses is shown in Figure 23, while Table 10 and Figure 24 summarize the DAB simulation results at V_out_ = 250 V and P_out_ = 4.125 kW.

### 2.3. Auxiliary PSFB Converter Design

The proposed OBC design provides the integration of a third conversion stage in order to supply the 12 V service battery from the HV-rail. Moreover, the exploitation of bidirectional devices and suitable control algorithms enables us to implement features such as the limp home and inverter HV DC-link capacitor’s precharge from the LV battery.

The most promising topologies to realize this high step-down DC/DC converter are as follows [17,34,35,36]:

- The phase-shift full bridge (PSFB) with center-tapped synchronous rectification;
- The phase-shift full bridge with full-bridge synchronous rectification;
- The current-doubler phase-shift full bridge;
- The active-clamp forward with synchronous rectification;
- The resonant LLC.

A one-fits-all solution does not exist, and the topology choice depends on the current and power levels as well as on the voltage ratio, part counts, complexity and cost. As anticipated in Figure 3, the center-tapped PSFB with synchronous rectification was selected for our design. In fact, it is a popular scheme for EV DC-DC converters, benefiting from a lower cost and lower complexity. In the boost operating mode, i.e., from the LV to the HV side, it appears as a current-fed push–pull DC-DC converter [37,38].

In Table 11, we recall the auxiliary DC-DC converter’s main specifications.

On the primary side, the designed PSFB exhibits a full bridge of top-side-cooled GS66508T GaN HEMTs (650 V, 30 A, 50 mΩ), driven by Allegro AHV85110 single-channel isolated drivers. On secondary side, two EPC2302 eGaN FETs (100 V, 101 A, 1.4 mΩ) in parallel are used per switch, driven by Texas Instruments UCC27611 single-channel drivers.

A high switching frequency (300 kHz) was selected to obtain very compact magnetics. The required transformer ratio can be calculated as follows [39]:

(15) $$n=\frac{N_{1}}{N_{2}}\leq \frac{V_{in,min}}{V_{out,\;nom}}\cdot D_{max}=\frac{240\;V}{12\;V}\cdot 0.7=14$$

The breaking voltage of the secondary side’s devices is then decided on the basis of

(16) $$V_{blocking\;max,\;sec}=\frac{{2\cdot V}_{in,max}}{n}=\frac{2\cdot 450\;V}{14}\approx 64\;V$$

We chose to use 100 V power switches to have a sufficient safety margin with respect to the well-known V_DS_ overvoltage spike issue of this topology due to the resonance between the output-rectifier parasitic capacitance and transformer leakage inductors [40]. An RCD snubber solution was adopted to minimize overvoltages in the synchronous rectifier devices.

In order to implement the peak-current mode control (PCMC), the magnetizing inductance of the transformer has to fulfill the condition [39]:

(17) $$L_{mag}\geq \;\frac{V_{in}\cdot \left(1-D_{typ}\right)\cdot n}{{∆I}_{Lout}\cdot 0.5\cdot 2f_{sw}}=\frac{360\;V\cdot \left(1-0.47\right)\cdot 14}{13.3\;A\cdot 0.5\cdot 600\;\mathrm{kHz}}\approx 670\;\mu H$$

where 2f_sw_ is the switching frequency of the output inductor and ΔI_Lout_ is the inductor ripple current (20% of the output current, which is equal to P_out_/V_out_ ≈ 67 Arms). PCMC guarantees a cycle-by-cycle check on the primary current of the PSFB transformer, preventing core saturation without the need for a bulky DC-blocking capacitor enhancing the power density. The output inductance can be computed as follows:

(18) $$L_{out}=\frac{V_{out}\cdot \;\left(1-D_{typ}\right)}{{∆I}_{Lout}\cdot 2f_{sw}}=\frac{12\;V\cdot \left(1-0.47\right)}{13.3\;A\cdot 600\;\mathrm{kHz}}\approx 0.8\;\mu H$$

In Table 12, the PSFB’s passive components are listed. A really compact size was achieved for the PSFB transformer: it was encapsulated in a 50 (L) × 34 (W) × 42 (H) [mm] potting box.

**Figure 25:**
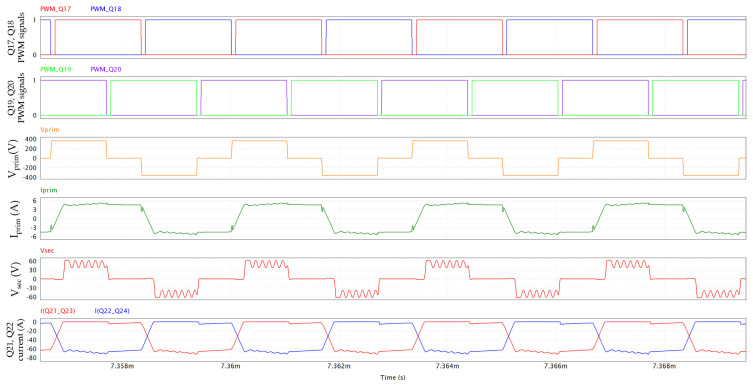
PSFB waveforms, including, from top to bottom, PWM signals of Q17 and Q18, PWM signals of Q19 and Q20, primary voltage, primary current, secondary voltage and Q21-Q23 and Q22-Q24 currents.

**Figure 26:**
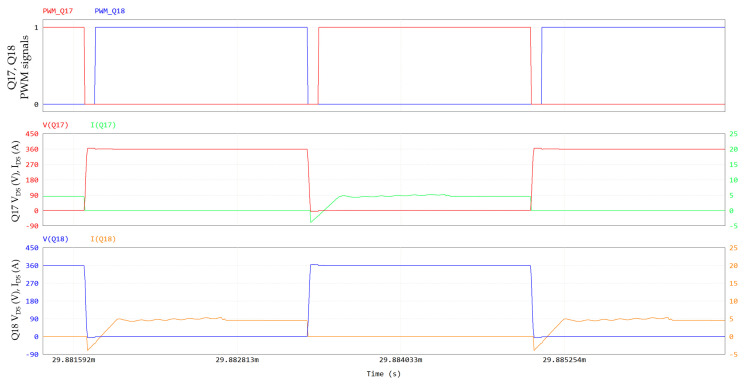
PSFB Q17 and Q18 ZVS turn-on: PWM signals of Q17 and Q18, V_DS_ and I_DS_ of Q17 and V_DS_ and I_DS_ of Q18. Y-axis of drain-source voltage is on left, Y-axis of drain-source current is on right.

**Figure 27:**
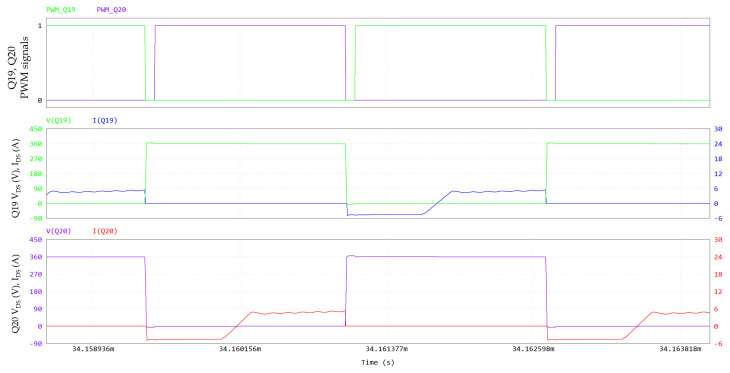
PSFB Q19 and Q20 ZVS turn-on: PWM signals of Q19 and Q20, V_DS_ and I_DS_ of Q19 and V_DS_ and I_DS_ of Q20. Y-axis of drain-source voltage is on left, Y-axis of drain-source current is on right.

In Figure 25, the PSFB waveforms (360 V/12 V; 800 W; 300 kHz; dead time = 80 ns; T_amb_ = 60 °C), simulated through PSIM, are shown. On the primary side, 650 V GaN HEMTs are driven with V_GS_ = 6/−3 V and R_G_ = 10/2 Ω, whereas 100 V eGaN FETs on the secondary side are driven with V_GS_ = 5/0 V and R_G_ = 1.6 Ω. The PSFB ZVS conditions can be calculated as reported in [35,36]. An external shim inductor is not used in our case, enhancing the power density. Figure 26 and Figure 27 display a ZVS turn-on for all the switches on the primary side.

The secondary voltage (V_sec_ in the fifth plot of Figure 25) corresponds to the V_DS_ of Q21-Q23 when Q22-Q24 are conducting and to −V_DS_ of Q22-Q24 when Q21-Q23 are conducting. The impact of the snubber is visible, maintaining the device’s drain-source voltages below 62 V, along with a typical ringing effect. The nominal blocking voltage of the secondary side’s devices under the mentioned operating conditions is about 51 V.

**Figure 28:**
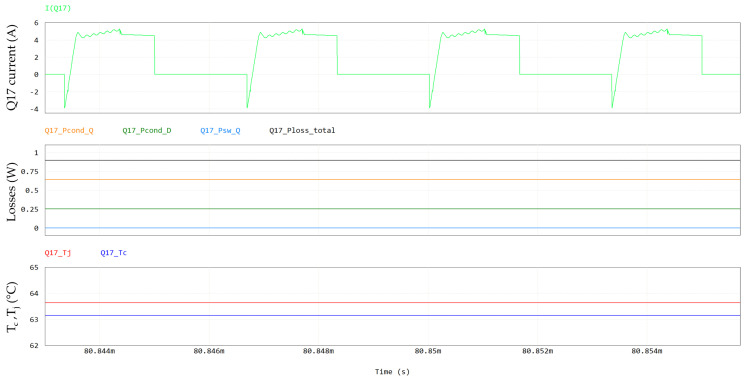
Q17 losses, including, from top to bottom, Q17 current, conduction losses (in orange), third-quadrant losses (in green), switching losses (in light blue), total losses (in black) and case (T_c_) and junction temperatures (T_j_).

**Figure 29:**
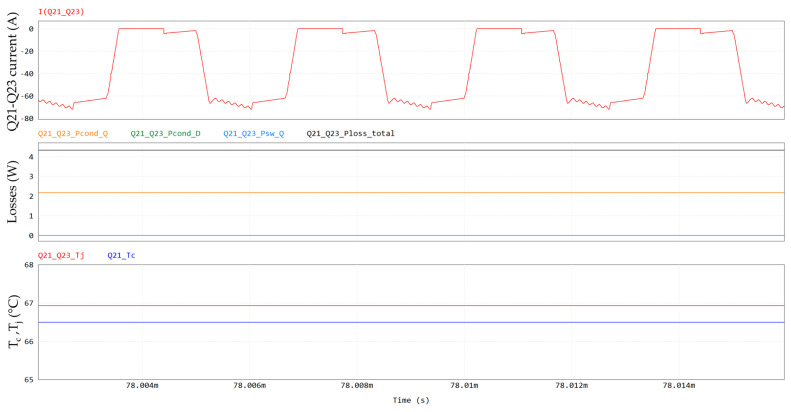
Q21/Q23 losses, including, from top to bottom, Q21-Q23 current, conduction losses (in orange), third-quadrant losses (in green), switching losses (in light blue), total losses (in black) and case and junction temperatures. Current in first plot and losses in second plot refer to power switch formed by parallel of Q21-Q23, whereas temperatures refer to individual device.

The Q17 (primary side) and Q21/Q23 (secondary side) currents, temperatures and power losses are shown in Figure 28 and Figure 29, respectively.

The custom-designed transformer (UI core) exploits Litz wire to reduce the skin effect. The simulation of power losses performed through ANSYS software results in 1.5 W for the core and 2.25 W and 6 W for winding losses on the primary and secondary sides, respectively. The power dissipation of the output inductor can be estimated as 1.33 W. The RCD snubber losses (two capacitors: 100 V, 220 nF; two resistors: 1 kΩ, 2 W) are computed as 4.83 W. The losses of the electrolytic capacitors can be quantified as 0.1 W, leading to negligible losses in terms of the total thermal budget.

**Figure 30:**
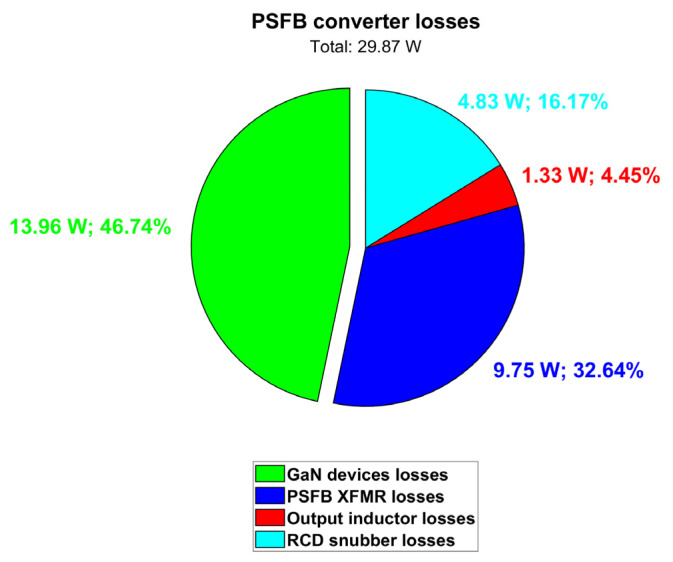
Contributions of PSFB converter losses (@ T_amb_ = 60 °C).

Table 13 and Figure 30 summarize the PSFB simulation results. The total dissipated power is quantified as 29.87 W and the PSFB efficiency turns out to be 96.40% (at a 60 °C coolant temperature).

### 2.4. Converter Control

**Figure 31:**
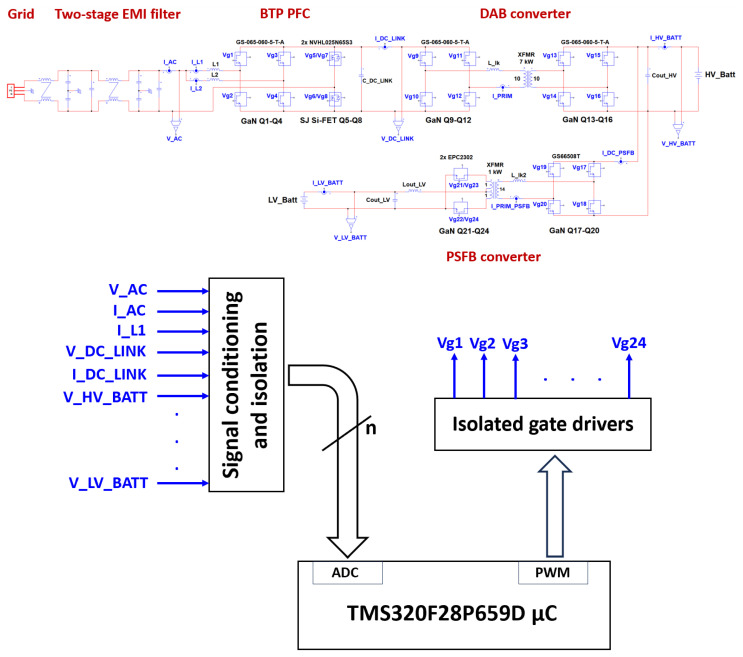
OBC’s simplified control architecture.

Figure 31, which recalls for convenience the overall system topology shown in Figure 3, describes at high level the OBC control architecture. Seven different currents and four different voltages are sensed by the means of isolated Hall-effect current sensors (Allegro ACS733KLATR-40AB-T) and reinforced isolated amplifiers (TI AMC3330DWER). The 32-bit 200 MHz real-time microcontroller TMS320F28P659D from the C2000 family of Texas Instruments is exploited to implement the control algorithms, along with CAN communications and other service tasks. Internal 12- and 16-bit ADCs are used for the digitalization of sensed signals. The µC generates 20 different gate PWM signals (since devices in parallel share the same PWM signal). Gate signal buffering and isolation are implemented by the gate drivers mentioned in the previous sections.

#### 2.4.1. Bridgeless Totem-Pole PFC Control

The BTP PFC control technique consists of two main feedback loops that regulate the output voltage (outer and slower loop) and the two input currents (inner and faster loops) where a sinusoidal shape is superimposed by sensing the L-N voltage for PFC purposes [11,40]. The bandwidth of the voltage and current regulators are set, respectively, to five time less than the grid frequency to avoid distortion, and a range of between a decade above the voltage bandwidth and a decade under the antiresonance frequency of the input EMI filter.

In order to improve the harmonic distortion and the power factor, three main techniques are implemented:

- A reduction in the current spikes during the zero-crossing, caused by the charge/discharge of the C_oss_ of the HF and LF devices, is obtained by implementing a soft-start procedure every half period [41,42,43].
- A PLL-SOGI filter is applied to the AC voltage-measured signal.
- An internal model compensator is applied to the input currents up to the ninth harmonic of the grid frequency.

Finally, a start-up procedure is designed to minimize the occurrence of the conduction of the diode-rectifier (placed parallel to the BTP to precharge the output capacitors) when the PWM is triggered to be on and the output voltage is tied to the voltage grid peak. Basically, the converter begins to be piloted a few moments after the peak value of the grid in order to boost the output voltage for almost an entire grid period, avoiding any diode being positively forward.

#### 2.4.2. DAB Control

The DAB is piloted in SPS modulation and controlled by a single feedback loop on the output voltage (similarly to [44]), where the reference power is retrieved and the phase-shift angle is forced to the converter. Since the load is an HV-battery, the regulator manages the amount of power by saturating to the maximum current on the level required by the battery or handled by the system.

Given the slow dynamic of the charging procedure compared to the control bandwidth, no feedforward terms are employed, avoiding regulator overshoot.

#### 2.4.3. PSFB Control

For the PSFB converter, the peak-current mode control (PCMC) is implemented, as described in [45,46]. This choice guarantees a cycle-by-cycle check on the primary current of the transformer, consequently preventing core saturation without the need for a bulky DC-blocking capacitor, enhancing the power density.

The outer voltage control is implemented by software and compensates for the plant (output capacitor) using a PI regulator, while the inner current control is entirely managed by dedicated hardware due to the bandwidth required (twice the switching frequency). The hardware resources are made available by F28P65x, which integrates a Comparator Subsystem (CMPSS) Type-6 and Enhanced PWM (ePWM) Type-5 specifically designed for such a control mode.

#### 2.4.4. Software

The entire system is managed by a TMS320F28P659D µC consisting of two separated cores. Given the different switching frequencies of BTP (130 kHz) and DAB/PSFB (300 kHz), the first converter relies on the first core, while the last two converters rely on the second. 

Concerning the execution timing, the code is organized into two main tasks: a fast task (30 kHz), where the control algorithms are executed, and a slow task (1 kHz), which is in charge of handling context conditions such as presence of HV plugs, enabling internal supplies, etc.

## 3. OBC Implementation and Validation Measurements

The OBC is implemented on an 8-layer PCB, optimizing the power density and wisely exploiting the layers as shielding ground planes for EMI minimization. All the high-frequency commutating GaN devices are placed on the bottom layer. The thermal pad of their top-side-cooled package is connected through a very-high-performance TIM (thermal interface material) to a custom-designed cold plate that spans beneath the entire PCB. The resulting thermal resistance between the GaN HEMT thermal pad and the cold plate is estimated as R_TH_ = 3 °C/W. The cold plate liquid is water glycol and the flow rate is 5 L/min; it is designed to maintain its surface beneath the GaN switches at 60 °C, when the ambient temperature is 50 °C (coolant temperature 55 °C). The PCB is properly shaped with internal and lateral slots so that all the custom magnetic components (i.e., the DAB transformer, PSFB transformer, and PFC choke inductors) encapsulated in the aluminum box are screwed directly to the cold plate to optimize power dissipation.

**Figure 32:**
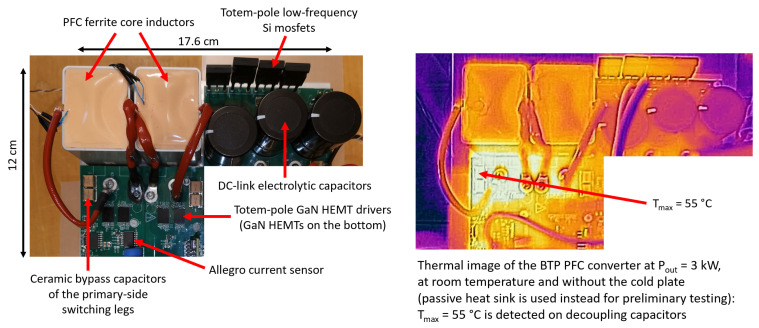
OBC prototype: BTP PFC converter and thermal image.

**Figure 33:**
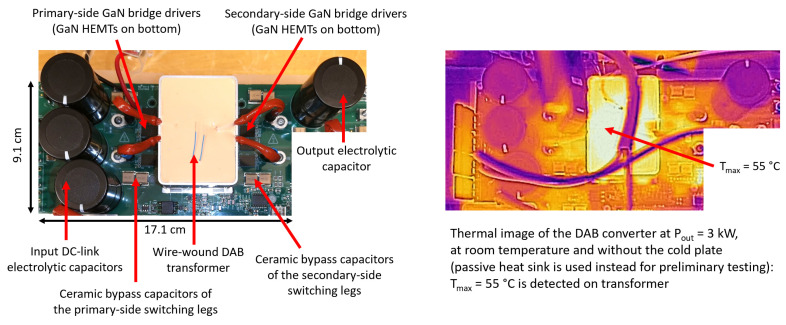
OBC prototype: DAB converter and thermal image.

**Figure 34:**
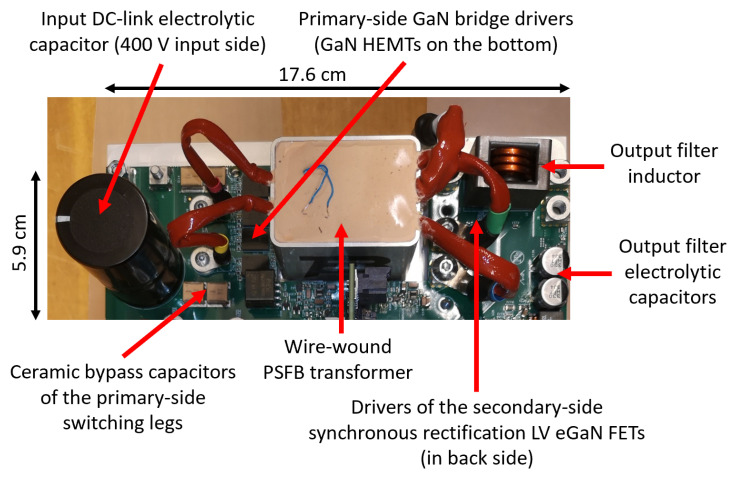
OBC prototype: PSFB converter.

In Figure 32, Figure 33 and Figure 34, the pictures of the different sections of the OBC are shown with some dimensional references. The dimensions of the different sections are also indicated in the figures. The space occupancy of the circuits is very limited, due to compact magnetics and capacitors enabled by the operation at a high switching frequency and the fast control. Moreover, the dimensions can be further reduced through the successive iteration of the PCB, since this first prototype is designed for the accessibility of probing for verification and debugging. The entire OBC PCB is enclosed in an aluminum box, integrating the cold plate and all the connectors to the grid and the batteries. 

At this stage, the first PCB prototype has been preliminarily, partially tested at room temperature, using a passive heat sink rather than the cold plate. The PFC section has been tested at full power (6.6 kW) with 230 Vrms, a 50 Hz grid voltage and a nominal 400 V output voltage (16.5 A output current). Also, the DAB converter has been tested at room temperature up to 4 kW with V_in_ = 400 V and a 380 V output voltage (10.53 A output current). A comparison between the measured and simulated performance of the entire high-voltage battery charging section (i.e., BTP PFC + DAB) at V_out_ = 380 V is provided in Table 14. The results were obtained by simulating the same measured conditions (the OBC mounted on the passive heatsink at ambient temperature). The estimation of the temperature of heatsink surface below GaN switches in this condition is between 50 and 60 °C.

**Figure 35:**
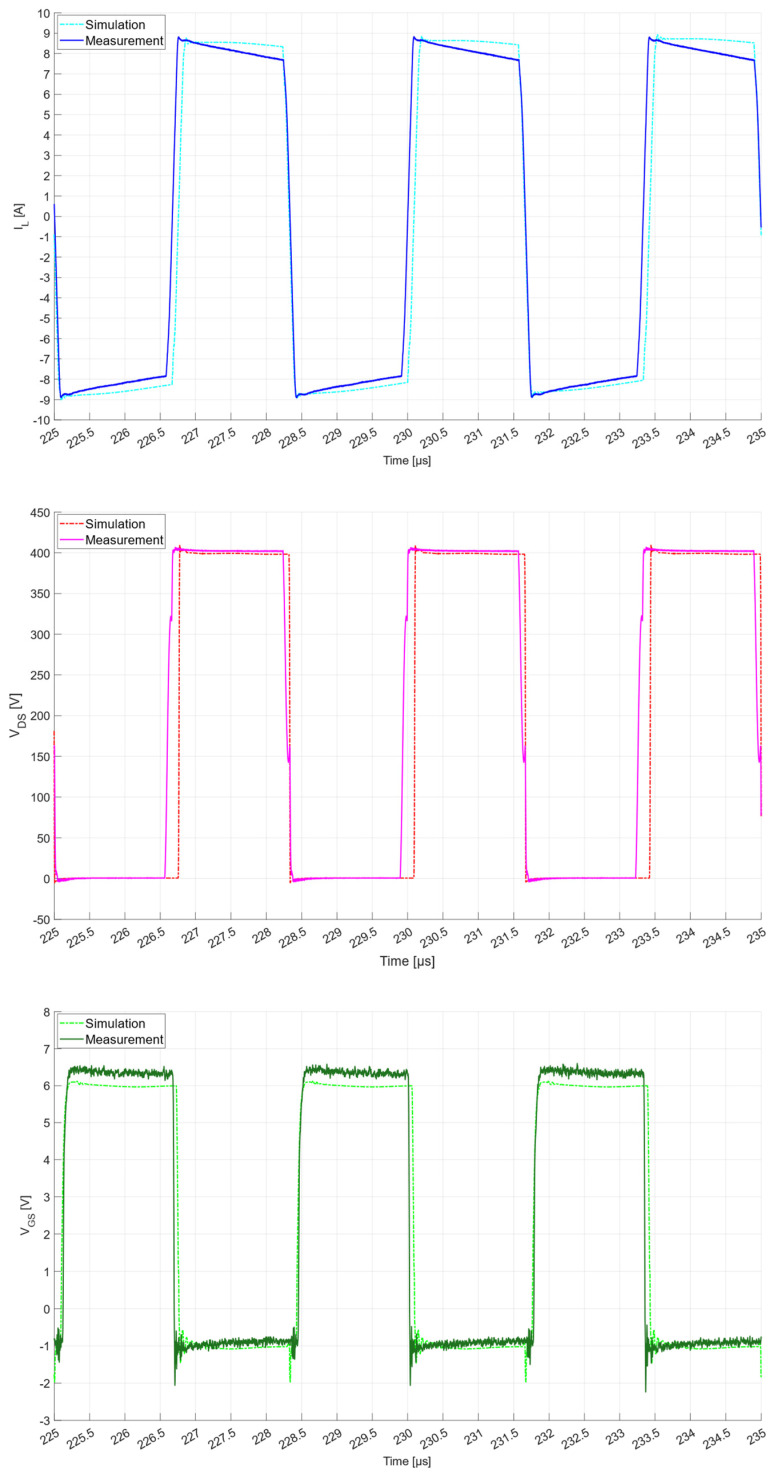
Series inductor current I_L_ and V_DS_ and V_GS_ of Q10 at 3.3 kW power level from post-layout simulation and scope (MSO-56 Tektronix) acquisition.

It is fair to notice that the converter performance computed by the preliminary measurement characterization is very close to the simulated values. Also, the thermal images of the converters confirmed the expected case temperatures of visible components such as magnetics, capacitors and gate drivers. Some examples of thermal images are shown in Figure 32 and Figure 33. Some measured waveforms at 3.3 kW, such as the DAB converter primary current and the V_DS_ and V_GS_ voltages of the DAB Q10 GaN HEMT, are shown in Figure 35, along with simulations. The waveforms are measured, exploiting wideband sensors (20 MHz Rogoski current probe and 100 MHz active differential voltage probe) and a digital oscilloscope (500 MHz MSO-56 Tektronix at 6.25 GS/s) that can capture data without filtering/attenuating any eventual high-frequency component (e.g., ringing) in the waveforms. The very clean waveforms without overshoots or ringing reveal an effective low-parasitic PCB design and the quality of the magnetics that exhibit extremely low parasitic capacitances. The simulation waveforms derive from the post-layout simulation of the DAB converter, i.e., also taking into account the S-parameters matrix which is the result of the EM simulation of the board, performed as described in [16], where a better trade-off between the third quadrant and switching losses [28] has been reported for our design in the case where V_GS,off_ = −1 V is implemented. 

The converter will be further tested at different operative regimes and temperature conditions and then will be encapsulated in a final metal box with an integrated cold plate for EMI pre-compliance characterization.

## 4. Conclusions

The comprehensive design of a 6.6 kW GaN-based OBC for automotive applications has been described. The OBC is composed of a monophase bridgeless totem-pole PFC stage operating at 130 kHz switching frequency that synthesizes at its output a 400 V DC with a very limited AC grid ripple. A DAB converter, operating at 300 kHz, regulates the DC- link voltage to the HV battery charging voltage in the range 200 V–450 V. In addition, an auxiliary DC-DC converter based on PSFB topology with synchronous rectification is used to connect the HV battery to the LV battery. All the converters are bidirectional, enabling very high flexibility. Detailed simulations enabled us to the estimate power loss distributions and overall performances by the means of accurate models of the components. The simulated performances at the maximum operative temperature of 60 °C are state-of-the-art. This was enabled by the exploitation of GaN switches (650 V in the high-voltage sections and 100 V switches in the low-voltage battery interface), custom designed high-performance magnetics, the accurate modelization and simulation of components, state-of-the-art controlling hardware and algorithms and accurate layout optimization for the minimization of parasitics. In this first prototype, the OBC exhibits a 2.2 kW/L volumetric power density, including the enclosure and the cooling system. This value can be largely improved in a future release that would not need accessibility to the testing points necessary for the first development. Preliminary characterization measurements on the first prototype are very close to the simulation results, indicating the effectiveness of our design and simulation approach.

## Figures and Tables

**Table 1:** Examples of commercial OBC design.

Manufacturer	PFC Topology	HV DC-DC Topology	Input Voltage (Vrms)	Output Voltage (V)	Nominal Power (kW)	Efficiency (%)	Power Density (kW/L)	Switching Devices
Texas Instruments [11]	2-ph interleaved BTP	CLLC	90–264	200–450	6.6	96.5 (@ 20 °C)	3.8 (open frame)	GaN power ICs
Navitas Semiconductor ^1^ [12]	2-ph interleaved BTP	CLLC	85–265	250–500	6.6	96.24 (@ 45 °C)	3.9	GaN power ICs
Delta-Q [18,19]	2-ph interleaved BTP	CLLC	85–265	200–450	6.6	96	2.26	GaN/SiC
Wolfspeed [20]	BTP	CLLC	90–265	250–450	6.6	96.5	3.3 (open frame)	SiC
Proposed prototype ^1^	2-ph interleaved BTP	DAB	90–264	200–450	6.6	96 (@ 60 °C)	2.2	GaN HEMTs

^1^ An auxiliary LV DC-DC converter is also integrated.

**Table 2:** OBC’s main specifications.

**PFC Stage**					**DC-DC HV Stage**					
**AC Grid Range**		**V_out,nominal_**		**P_out,nominal_**	**V_in,nominal_**	**V_out,range_**	**V_out,nominal_**	**I_out,nominal_**	**P_out,nominal_**	**P_out,max_**
90–264 Vrms		400 V		6.6 kW	400 V	200–450 V	400 V	16.5 A	6.6 kW	7 kW
**DC-DC LV Stage**							**Additional Requirements**			
**V_in,range_**	**V_in,nominal_**		**V_out,range_**	**V_out,nominal_**	**P_out,nominal_**	**P_out,max_**	Bidirectional power flow CISPR 32/EN 55022/32 Class B compliance			
240–450 V	360 V		10–16 V	12 V	800 W	1 kW				

**Table 3:** Datasheet parameters of GaN HEMT GS-065-060-5-T-A.

**V_DS_**	**I_DS_** **(@ Tc = 25 °C)**		**I_DS_** **(@ Tc = 100 °C)**	**R_DS,on_** **(@ Tj = 25 °C)**		**R_DS,on_** **(@ Tj = 150 °C)**
650 V	60 A		41 A	25 mΩ		65 mΩ
**C_iss_** **(@ 400 V)**	**C_oss_** **(@ 400 V)**		**C_gd_** **(@ 400 V)**	**Q_gd_** **(@ 400 V)**		**Q_g_** **(@ 400 V)**
516 pF	127 pF		2.4 pF	4.1 nC		14 nC
**E_on_, E_off_, E_oss_** **(@ 400 V, 20 A, R_G_ = 10/2 Ω, V_GS_ = 6/−3 V, Tj = 25 °C)**						
117 µJ		17.2 µJ			17 µJ	
**Package inductances L_g_, L_d_, L_s_** (from Pspice level 3 model)						
4 nH		0.2 nH			0.3 nH	

**Table 4:** PFC’s passive components.

Passive Component	PN	Quantity	Parameters
PFC inductor	Bourns custom design	2×	L = 60 µH (@ 1 V, 100 kHz) R_DC_ = 22 mΩ Saturation Current: 20% Roll off
		3×	V_DC_ = 500 V C = 390 µF
Electrolytic capacitor	Kemet ALA7DA391CF500		ESR = 481.2 mΩ (@ 20 °C, 10 kHz) ESL = 20 nH I_crms_ = 4.12 Arms (@ 85 °C, 10 kHz)
			V_DC_ = 500 V C = 1 µF
Ceramic capacitor	TDK B58031U5105M062	2× for each GaN leg	ESR = 12 mΩ (@ 0 V_DC_, 0.5 Vrms, 25 °C, 1 MHz) ESL = 3 nH I_crms_ = 11 Arms (@ 85 °C, 100 kHz)

**Table 5:** The RMS and mean values of the PFC waveforms at the output section.

Quantity	RMS Value	Mean Value
V_out_	400.37 Vrms	400.06 V
I_out_	16.52 Arms	16.50 A
I_C,DC-link_	13.32 Arms	≈0 A

**Table 6:** The main frequency components of the PFC waveforms at the output section.

Quantity	Amplitude (0 Hz)	Amplitude (100 Hz)	Amplitude (260 kHz)
V_out_	400 Vpk	22.04 Vpk	0.55 Vpk
I_out_	16.50 Apk	0.91 Apk	0.023 Apk
I_C,DC-link_	≈0 Apk	16 Apk	4.41 Apk

**Table 7:** PFC simulation results at T_amb_ = 60 °C; V_in_ = 240 Vrms; V_out_ = 400 V; and P_out_ = 6.6 kW.

Quantity	Value
Line current	28 Arms
Boost inductor current	14.27 Arms 23.17 A peak value 12.94 A_pk-pk_ = ΔI_max_
GaN HEMT current	10.07 Arms
Si mosfet current	9.90 Arms
DC-link capacitor bank current	13.32 Arms
GaN HEMTs temperature	T_c_ = 96 °C, T_j_ = 100 °C
Si mosfets temperature	T_c_ = 68 °C, T_j_ = 69 °C
Inductor copper losses	(2×) 6 W
GaN HEMTs losses	(4×) 12 W
Si mosfets losses	(4×) 3 W
DC-link capacitor bank losses	14.79 W
Total losses	86.79 W
V_out_	400 V
V_out_ voltage ripple	45 V_pk-pk_
I_out_	16.5 Arms
P_out_	6600 W
P_in_	6686.79 W
Efficiency	98.70%
THD	6%
PF	0.996

**Table 8:** DAB’s passive components.

Passive Component	PN	Quantity	Parameters	
DAB XFMR	Bourns custom design	1	C_p,s_ = 27.2 pF C_ww_ = 43.3 pF R_DCp,s_ = 9.4 mΩ	L_lk_ = 6 uH L_mag_ = 301.6 uH Turns ratio = 10:10
		3× in the DC-link section 1× in the output section	VDC = 500 V C = 390 uF	
Electrolytic capacitor	Kemet ALA7DA391CF500		ESR = 481.2 mΩ (@ 20 °C, 10 kHz) ESL = 20 nH I_crms_ = 4.12 Arms (@ 85 °C, 10 kHz)	
			VDC = 500 V C = 1 uF	
Ceramic capacitor	TDK B58031U5105M062	2× for each GaN leg	ESR = 12 mΩ (@ 0 VDC, 0.5 Vrms, 25 °C, 1 MHz) ESL = 3 nH I_crms_ = 11 Arms (@ 85 °C, 100 kHz)	

**Table 9:** DAB simulation results at T_amb_ = 60 °C; V_in_ = 400 V; V_out_ = 400 V; P_out_ = 6.6 kW; f_sw_ = 300 kHz.

Quantity	Value
XFMR current at primary	19.21 Arms 21.21 A peak value
XFMR current at secondary	19.43 Arms 21.77 A peak value
Current of GaN HEMT at primary	13.54 Arms 21.21 A peak value
Current of GaN HEMT at secondary	13.68 Arms 21.77 A peak value
DC-link capacitor bank current	3.54 Arms
Output capacitor current	1.47 Arms
Temperature of GaN HEMTs at primary	T_c_ = 110 °C, T_j_ = 116 °C
Temperature of GaN HEMTs at secondary	T_c_ = 117 °C, T_j_ = 123 °C
DAB XFMR losses	17.7 W
Losses of GaN HEMTs at primary	(4×) 17 W
Losses of GaN HEMTs at secondary	(4×) 19 W
DC-link capacitor bank losses	1.04 W
Output capacitor losses	0.54 W
Total losses	163.28 W
V_out_	400 V
V_out_ voltage ripple	1.58 V_pk-pk_
I_out_	16.5 Arms
P_out_	6600 W
P_in_	6763.28 W
Efficiency	97.59%

**Table 10:** DAB simulation results at T_amb_ = 60 °C; V_in_ = 400 V; V_out_ = 250 V; P_out_ = 4.125 kW; and f_sw_ = 300 kHz.

Quantity	Value
XFMR current at primary	19.17 Arms 33.63 A peak value
XFMR current at secondary	19.06 Arms −33.44 A negative peak value
Current of GaN HEMT at primary	13.45 Arms 33.63 A peak value
Current of GaN HEMT at secondary	13.77 Arms −34.44 A negative peak value
DC-link capacitor bank current	7.38 Arms
Output capacitor current	2.10 Arms
Temperature of GaN HEMTs at primary	T_c_ = 120 °C, T_j_ = 127 °C
Temperature of GaN HEMTs at secondary	T_c_ = 107 °C, T_j_ = 113 °C
DAB XFMR losses	13.91 W
Losses of GaN HEMTs at primary	(4×) 20 W
Losses of GaN HEMTs at secondary	(4×) 16.5 W
DC-link capacitor bank losses	4.54 W
Output capacitor losses	1.10 W
Total losses	165.55 W
V_out_	250 V
V_out_ voltage ripple	1.75 V_pk-pk_
I_out_	16.5 Arms
P_out_	4125 W
P_in_	4290.55 W
Efficiency	96.14%

**Table 11:** DC-DC LV converter main specifications.

DC-DC LV Stage					
V_in,range_	V_in,nominal_	V_out,range_	V_out,nominal_	P_out,nominal_	P_out,max_
240–450 V	360 V	10–16 V	12 V	800 W	1 kW

**Table 12:** PSFB’s passive components.

Passive Component	PN	Quantity	Parameters
PSFB XFMR	Bourns custom design	1	L_mag_ = 838.6 µH (@ 100 kHz) L_lk_ = 9.3 µH (@ 100 kHz) R_DC,p_ = 23.6 mΩ R_DC,s1,s2_ = 1.2 mΩ C_p,s_ = 14 pF C_ww_ = 87 pF Turns ratio = 14:1:1
Electrolytic input capacitor	Kemet ALA7DA391CF500	1	VDC = 500 V C = 390 µF ESR = 481.2 mΩ (@ 20 °C, 10 kHz) ESL = 20 nH I_crms_ = 4.12 Arms (@ 85 °C, 10 kHz)
Ceramic capacitor	TDK B58031U5105M062	2× for each GaN leg at primary	V_DC_ = 500 V C = 1 µF ESR = 12 mΩ (@ 0 V_DC_, 0.5 Vrms, 25 °C, 1 MHz) ESL = 3 nH I_crms_ = 11 Arms (@ 85 °C, 100 kHz)
Output inductor	Vishay IHDM1107BBEV1R1M20	1	L = 1.1 µH (@ 100 kHz, 0.25 V, 0 A) DCR = 0.30 mΩ (@25 °C) Saturation current = 301 A (@ 30% of L drop)
Electrolytic output capacitor	Panasonic EEEFT1H331AV	2×	V_DC_ = 50 V C = 330 µF ESR = 120 mΩ (@ 20 °C, 100 kHz) I_crms_ = 0.9 Arms (@ 105 °C, 100 kHz)
Ceramic output capacitor	Murata GRM32ER7YA106KA12K	5×	V_DC_ = 35 V C = 10 µF ESR = 2 mΩ (@ 0 V_DC_, 25 °C, 1 MHz)

**Table 13:** PSFB simulation results at T_amb_ = 60 °C; V_in_ = 360 V; V_out_ = 12 V; P_out_ = 800 W; and f_sw_ = 300 kHz.

Quantity	Value
XFMR current at primary	4.47 Arms 5.40 A peak value
XFMR current at secondary	45.54 Arms −72.40 A negative peak value
Current of GaN HEMT at primary	3.15 Arms 5.40 A peak value
Current of GaN switch at secondary	45.54 Arms −72.40 A negative peak value
Input electrolytic capacitor current	0.56 Arms
Output capacitor bank current	2 Arms
Output inductor current	66.67 Arms 71.15 A peak value 9.45 A_pk-pk_ = ΔI_max_
Temperature of GaN HEMTs at primary	T_c_ = 63.1 °C, T_j_ = 63.6 °C
Temperature of eGaN FETs at secondary	T_c_ = 66.3 °C, T_j_ = 66.9 °C
PSFB XFMR losses	9.75 W
Losses of GaN HEMTs at primary	(4×) 1.3 W
Losses of eGaN FETs at secondary	(4×) 2.19 W
Output inductor losses	1.33 W
RCD snubber losses	4.83 W
Total losses	29.87 W
V_out_	12 V
V_out_ voltage ripple	0.43 V_pk-pk_
I_out_	66.67 Arms
P_out_	800 W
P_in_	829.87 W
Efficiency	96.40%

**Table 14:** Comparison between simulation and preliminary characterization tests of entire high-voltage battery charging section (i.e., BTP PFC + DAB) at V_out_ = 380 V.

Quantity	Measurement	Simulation	Meas.	Sim.	Meas.	Sim.	Meas.	Sim.
P_out_	2 kW		2.5 kW		3 kW		4 kW	
Efficiency	95.53%	95.22%	96.82%	96.31%	96%	96.5%	96.45%	97%
THD	4.29%	4.35%	2.96%	3.11%	2.75%	2.84%	2.63%	2.77%
PF	99.91%	99.87%	99.96%	99.90%	99.96%	99.91%	99.94%	99.89%

## Data Availability

The data presented in this study are available in the article.

## Abbreviations

AC	alternating current
BEV	battery electric vehicle
BOM	bill of materials
BTP	bridgeless totem-pole
CAN	controller area network
CCM	continuous conduction mode
CMPSS	comparator subsystem
CrCM	critical conduction mode
DAB	dual active bridge
DC	direct current
DCM	discontinuous conduction mode
DCR	direct current resistance
EM	electromagnetic
ESL	equivalent series inductance
ESR	equivalent series resistance
EU	European Union
EV	electric vehicle
FET	field-effect transistor
GaN	gallium nitride
G2V	grid-to-vehicle
HEMT	high-electron-mobility transistor
HF	high-frequency
HV	high-voltage
IC	integrated circuit
LF	low-frequency
LV	low-voltage
L-N	line-neutral
OBC	on-board charger
PCB	printed circuit board
PCMC	peak-current mode control
PF	power factor
PFC	power factor correction
PI	proportional integral
PLL	phase-locked loop
PSFB	phase-shifted full-bridge
PWM	pulse width modulation
QFNL	quad flat no-lead
Si	silicon
SiC	silicon carbide
SOGI	second-order generalized integrator
SPS	single-phase-shift
THD	total harmonic distortion
TO	transistor outline
USA	United States of America
V2G	vehicle-to-grid
V2L	vehicle-to-load
WBG	wide-band-gap
XFMR	transformer
ZVS	zero-voltage switching
µC	microcontroller
